# The Effect of Growth Medium Strength on Minimum Inhibitory Concentrations of Tannins and Tannin Extracts against *E. coli*

**DOI:** 10.3390/molecules25122947

**Published:** 2020-06-26

**Authors:** Sara Štumpf, Gregor Hostnik, Mateja Primožič, Maja Leitgeb, Juha-Pekka Salminen, Urban Bren

**Affiliations:** 1 Faculty of Chemistry and Chemical Engineering, University of Maribor, 2000 Maribor, Slovenia; sara.stumpf@um.si (S.Š.); gregor.hostnik@um.si (G.H.); mateja.primozic@um.si (M.P.); maja.leitgeb@um.si (M.L.); 2 Faculty of Medicine, University of Maribor, 2000 Maribor, Slovenia; 3 Natural Chemistry Research Group, Department of Chemistry, University of Turku, 20014 Turku, Finland; j-p.salminen@utu.fi; 4 Faculty of Mathematics, Natural Sciences and Information Technologies, University of Primorska, 6000 Koper, Slovenia

**Keywords:** tannins, vescalagin, castalagin, *E. coli*, antibacterial properties, minimum inhibitory concentration (MIC)

## Abstract

In this study the effect of growth medium strength on the minimum inhibitory concentration (MIC) of different tannins and tannin extracts against *Escherichia coli* was systematically investigated for the first time. Three pure compounds (vescalagin, castalagin and gallic acid) and five extracts (chestnut, quebracho, mimosa, Colistizer and tannic acid) were studied. Broth microdilution was assayed and bacteria were grown using different growth medium strengths varying from half to double the concentration recommended by the producer. MICs were determined using the iodonitrotetrazolium chloride (INT) dye or turbidity measurements. It was observed that MIC values depend on the growth medium strength. With an increase in the growth medium concentration MIC values rose roughly linearly for all samples, while their relative order remained unchanged, indicating that a direct interaction of tannins with growth medium nutrients represents the likely source of their antimicrobial activity. Understanding the effect of growth medium strength can finally yield a plausible explanation for the observed variation in MIC values reported in the scientific literature as well as provide help in planning proper applications of tannins in the livestock production.

## 1. Introduction

The discovery of antibiotics represents one of the greatest breakthroughs of modern medicine. They are often used for treating diseases of domestic animals. In the last decades, the usage of antibiotics in animal breeding became too widespread as they were added for non-therapeutic purposes as well, since it was discovered that they improve growth rates and consequently reduce costs of production. The use of antibiotics for non-therapeutic purposes as growth-promoting factors in the European Union has been banned after 2006 [1], while it remains allowed in several parts of the world. This widespread application of antibiotics led to an increase in antimicrobial resistance in animal microbiota and later also generated a transfer of resistance genes from animal to human bacteria strains, which together with misprescription and overprescription of antibiotics to patients caused the development of multiple drug-resistant pathogenic bacteria [2,3,4,5,6,7]. Without the application of antibiotics, the incidence of infectious diseases may increase and the animal production decrease, therefore, the usage of alternative prophylactic substances to control bacterial growth in the digestive tract and feed of animals are urgently needed [2]. Research of plant extracts as feed additives has gained popularity in recent years, because it has provided several promising results in the control of bacterial growth and the growth performance of animals [8]. So nowadays, plant-derived products or extracts are being routinely used as feed additives for livestock in order to at least partly substitute antibiotics.

**Figure 1:**
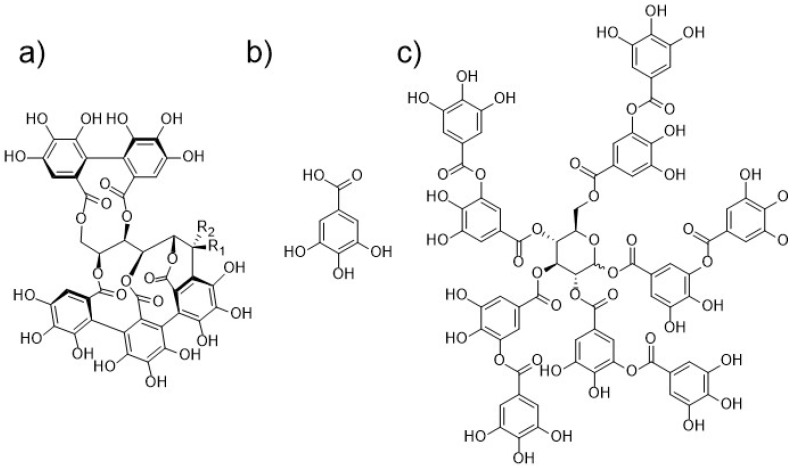
Chemical structures of (**a**) diastereoisomers castalagin (R_1_ = H, R_2_ = OH) and vescalagin (R_1_ = OH, R_2_ = H), (**b**) gallic acid, (**c**) decagalloylglucose, one of the gallotannins typically present in tannic acid.

One group of such compounds represent tannins, which are secondary plant metabolites from the class of polyphenols that are found in a variety of plant tissues as well as in different foods and feeds [9,10,11]. They are traditionally defined, as proposed by Bate-Smith and Swain, as water-soluble phenolic compounds having molar mass from 500 to 3000 g/mol. These substances are also capable of precipitating gelatin from solution, which is called astringency [12,13]. Because this definition does not include all substances, that should be classified as tannins (e.g., compounds of a higher molar mass), several alternative definitions have also been suggested [14,15]. Tannins of terrestrial plants are generally divided into two groups, hydrolysable tannins, and proanthocyanidins (condensed tannins) [14]. As is evident from their name, hydrolysable tannins can be hydrolysed into smaller compounds, e.g., gallic and ellagic acid. Chemical structures of castalagin, vescalagin, gallic acid, and decagalloylglucose are depicted in Figure 1. Proanthocyanidins are on the other hand typically composed of different structures of flavan-3-ol type (e.g., catechin, epicatechin, gallocatechin). Tannins are well known for their ability to form chelates with several metal ions [16]. Modern studies have shown that tannins also possess various health-promoting properties. They exhibit antibacterial, antiviral, antiparasitic, antioxidative, anti-inflammatory and anticarcinogenic activities [9,17,18].

*Escherichia coli* represents probably the most investigated microorganism and the strain used, K12, represents a lab strain, largely used in Molecular Biology due to the availability of a wide range of tools for its engineering. Due to its wide availability, MIC of tannic acid against *E. coli* was determined by Henis et al. [19], Taguri et al. [20], and Chung et al. [17,18]. For the same reason, MIC of gallic acid against *E. coli* was also measured in several studies [19,21,22]. On the contrary, vescalagin and castalagin are not so easily obtainable, but MIC values of both compounds against various microorganisms are also reported in the scientific literature [20,23,24,25]. MIC of castalagin against *E. coli* was for example determined by Taguri et al. [20]. The interpretation of the results obtained from these studies is difficult since MIC values vary significantly between studies. In the case of the tannic acid this spread can be mostly ascribed to the different sources and therefore different compositions of tannic acid [15]. In the case of pure compounds this spread of results can be partially ascribed to different experimental procedures of MIC determination (e.g., microdilution assay experiments typically yield lower MIC values than agar dilution) [26]. The obtained MIC values may also depend on inoculum size [27], while the effect of the applied bacterial strain is also far from negligible [20]. Another significant effect that influences the microbial growth could also be the variation of the growth medium composition [28,29], but a systematic study addressing effects of growth media strength on MICs of tannins is to the best of our knowledge still missing.

Several mechanisms for tannin inhibition of bacterial growth are proposed, which are usually classified into three main groups [11] (i) interaction of tannins with bacterial and substrate proteins [30,31]; (ii) interaction with bacterial cell wall plasma membrane [32]; and (iii) chelation of metal ions [30,33,34]. Considering the proposed tannin mechanisms of antibacterial action, the growth media composition could significantly influence the MIC values [35]. The likely source of this effect lies in the interactions of tannins with the components of the growth media, either by disabling the tannins from further direct action against bacteria or by preventing bacteria from accessing these components. In the case that the main antibacterial mechanism would, for example, represent the chelation of metal ions, a richer growth medium would result in higher metal ion concentration and therefore an increased amount of tannins would be needed to chelate all the ions thereby making them unavailable to the bacteria.

Tetracyclines form a class of antibiotics with a wide range of antibacterial activities. They act against Gram-negative and Gram-positive pathogens. The mechanism of antibacterial action of tetracyclines lies in their binding to 30S ribosomes and in inhibiting the protein synthesis by preventing the access of aminoacyl tRNA to the acceptor site on the ribosome [36]. They are commonly used in livestock production, but they are also of great importance in human medicine [37]. With the extensive use of tetracyclines in livestock production, bacteria developed tetracycline specific resistance mechanisms [36]. Although a certain dependence of MIC values on the growth media strength is still expected for tetracyclines with specific antibacterial mechanisms (i.e., they target specific metabolic pathways), this dependence should be much less pronounced than in the case of tannins. Because of their antimicrobial activity, tannins could eventually at least partially replace antibiotics as antibacterial agents added to the domestic animal feed.

This study aimed to gain additional insight into mechanism of antibacterial activity of hydrolysable tannins by examining the effect of growth media strength on MIC values of pure hydrolysable tannins (castalagin, vescalagin), a common hydrolysable tannin building block (gallic acid) and extracts containing hydrolysable tannins (chestnut, and tannic acid) against *E. coli*. Furthermore we showed that dependence of MIC values on media strength is valid not only for hydrolysable tannins and pure compounds by determining MIC values of crude commercial extracts (quebracho, mimosa, and Colistizer). Therefore, *E. coli* growth media of four different concentrations, ranging from half to double the concentration recommended by the producer, were prepared and the dependence of tannin MIC values on the growth media strength was determined.

## 2. Results and Discussion

### 2.1. Identification of Isolated Vescalagin and Castalagin from the Chestnut Extract

The sample identification was performed by comparing the retention time, UV/Vis spectrum, MS, and MS/MS data to the standard. The purity of the obtained product was determined by UPLC chromatography coupled with DAD and mass spectroscopy. Ammounts of obtained vescalagin and castalagin were 0.95 and 0.94 g, respectively. Chromatographic purity of the former was 97.0% whereas chromatographic purity of the latter was 95.9%. The minor impurities in vescalagin and castalagin were xylose/lyxose derivative of vescalagin (1066 Da) and gallic acid derivative of vescalagin/castalagin (1086 Da), respectively. Obtained 1H NMR spectra of vescalagin and castalagin are available in Supplementary Material Figures S1 and S2, respectively.

### 2.2. Determination of MICs for Selected Tannins against E. coli

**Figure 2:**
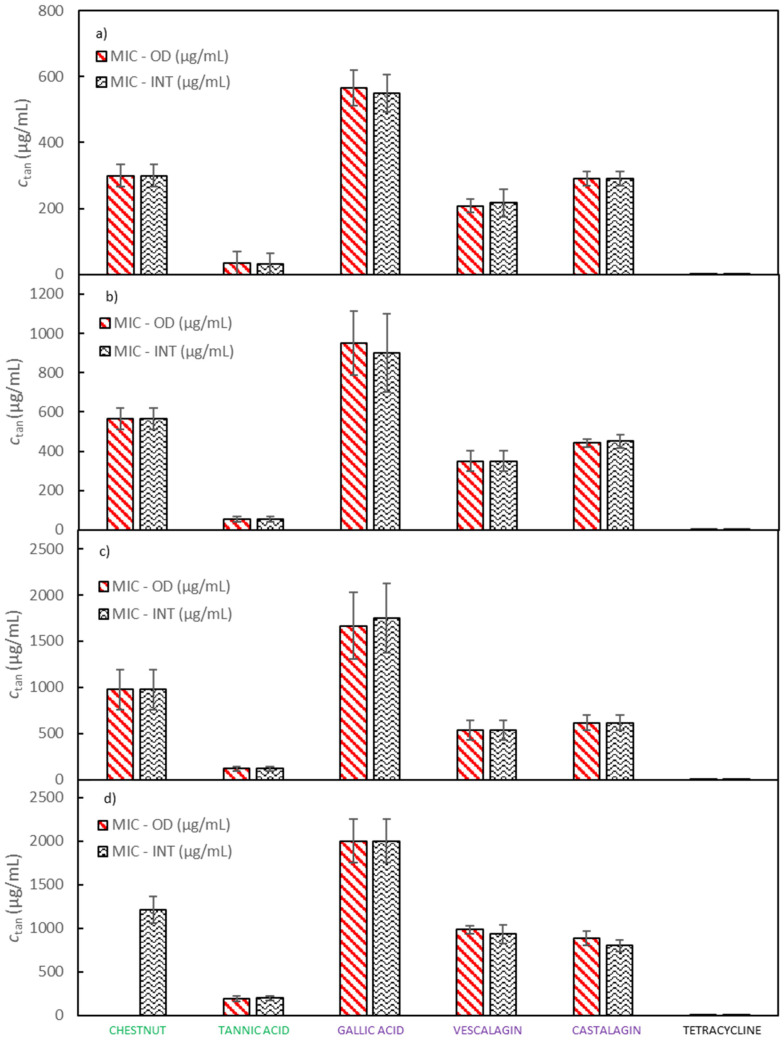
A comparison of MIC values, determined by measurement of the OD and by addition of the INT dye at (**a**) half the concentration of *E. coli* growth medium recommended by the producer, (**b**) the concentration of *E. coli* growth medium recommended by the producer, (**c**) one and a half the concentration of *E. coli* growth medium recommended by the producer and (**d**) double the concentration of *E. coli* growth medium recommended by the producer. Error bars represent 95% confidence interval.

MICs for extracts of quebracho, chestnut and mimosa, Colistizer, and tannic acid, as well as for pure compounds of gallic acid, castalagin, and vescalagin against *E. coli* were determined with two complementary methods, by the OD measurement and by the INT method. Experiments were carried out in six runs; thus, average MIC values and 95% confidence intervals for the chestnut extract, vescalagin, castalagin, gallic acid, tannic acid and tetracycline are depicted in Figure 2 and for the quebracho, mimosa and Colistizer extracts in Supplementary Material Figure S3 while numerical MICs with 95% confidence intervals are reported in Supplementary Material Tables S1 and S2. In Figure 2 a comparison of MIC values determined with the measurement of OD and with the addition of INT dye is displayed. It can be observed that both sets of MIC values are very similar, so the method of determination did not significantly affect the results. The advantage of OD measurement lies in enabling us to obtain more information about the bacterial growth. For instance, generation times and durations of the lag phase can be observed along with MIC values. The main weakness of this method lies in its fundamental principle of measurement. Since the method measures the turbidity of the sample, that is produced by bacterial cells, it is difficult or even impossible to follow bacterial growth in samples which are turbid themselves. Absorption of light at a wavelength at which turbidity is followed may also represent an interference. On the other hand, the determination of MIC using INT dye is relatively simple to perform and turbidity as well as coloration of a studied compound mostly do not affect the accuracy of the measurements. In the growth medium of double the concentration recommended by the producer, the tested solutions of chestnut, quebracho and mimosa extracts as well as Colistizer were not sufficiently soluble. Therefore, MIC values for these extracts using the OD method are not shown. Gallic acid, vescalagin, and castalagin represent the main components of the chestnut extract and are well soluble in aqueous media as is tannic acid. Also, MIC values for pure compounds, determined in the growth medium of double the concentration recommended by the producer, do not vary significantly from the trend, obtained in growth media of lower concentrations. However, MIC values for all studied extracts and pure compounds using the INT dye method were successfully determined.

All tested samples show promising antibacterial activities. For gallic acid the highest MIC value was determined, while from all tested samples tannic acid exhibits the best antibacterial properties by inhibiting the growth of *E. coli* with the lowest MIC value. MIC values for other samples lie between the values of gallic and tannic acid and their relative order remains unaffected by the growth medium strength. MIC values of chestnut, quebracho and mimosa extracts are similar to each other as are MIC values of castalagin and vescalagin. MIC of tetracycline was around 1000 times lower than MIC of any other tested compound regardless of the method and media concentration used. Colistizer, a commercial extract allegedly containing tannic acid, had a significantly higher MIC than the tannic acid bought from Sigma-Aldrich. This discrepancy may be to a great extent explained by the fact, that the composition of tannic acid from different sources varies significantly. Vescalagin and castalagin represent diastereoisomers and, therefore it is not surprising, that due to the structural similarity their MIC values are very similar as well. MIC values of vescalagin and castalagin are also lower than the MIC values of the original chestnut extract. This agrees well with the fact that vescalagin and castalagin form important constituents of the chestnut extract and confirms that both compounds contribute significantly to chestnut’s antimicrobial activity. However, both compounds represent about 10% of the extract mass cumulatively. Consequently, if these two compounds were the only source of antimicrobial activity, their expected MIC values would have to be an order of magnitude smaller than MIC values of the chestnut extract. Because MIC values of vescalagin and castalagin are only approximately 20% lower than MIC values of the chestnut extract, other components of the chestnut extract are likely to show significant antimicrobial activity as well [38].

Gallic and tannic acid have been already examined in several studies. Henis et al. [19] showed that the MIC value of tannic acid is significantly lower than the MIC value of gallic acid, which agrees well with our results. Antibacterial activity of tannic acid against many intestinal bacteria (*Clostridium perfringens*, *Clostridium clostridiiforme*, *E. coli*, *Salmonella typhimurium*, *Enterobacter cloacae*, etc.) was also proven by Chung et al. [17,18]. In these studies [17,18], gallic acid did not display any antibacterial activity on *E. coli*, while in our study gallic acid, although showing the weakest antibacterial activity, was still able to prevent bacterial growth. Some additional care should be taken when comparing these results. The highest gallic acid concentration, which Chung et al. [17] examined in their study, was 1000 μg/mL, while in our study the determined MIC values of gallic acid ranged between 550 and 2000 μg/mL, for growth media of the lowest and the highest strength, respectively. Their MIC values reported for tannic acid are of the same order of magnitude as observed in our study. Even if tannic acid molecules can hydrolyse into several gallic acid molecules, the significant difference in MIC values of both compounds can still be observed. The antimicrobial activity of tannic acid is assumed to be the consequence of its strong iron-binding ability [18]. Tannic acid possesses a much stronger iron complexation capability than gallic acid and can, therefore, form complexes with iron from bacterial growth media more successfully [18]. Because aerobic microorganisms require iron for many functions (e.g., reduction of ribonucleotide DNA precursors, heme formation, etc.), the result could be a strong inhibition of bacterial growth [17]. Of course, tannins may exhibit various other mechanisms of antimicrobial activity.

Min et al. [39] examined the effect of tannin extracts on in vitro and in vivo growth of *E. coli* O157:H7. In the in vitro study, where bacterial growth inhibition was determined by measuring OD at a wavelength of 600 nm, commercially available tannin extracts of chestnut and mimosa were tested. Both extracts exhibited bacteriostatic and bactericidal effects on the *E. coli* growth. They also observed that mimosa extract exerted a higher bacteriostatic activity while chestnut extract exerted a greater bactericidal activity. In our study, both extracts demonstrated antibacterial activity and the mimosa extract indeed exhibited slightly lower MIC values than the chestnut extract, which is in agreement with the results obtained by Min et al. [39]. Comparison is however additionally complicated by the fact that extracts of the same plant species may possess different concentrations of active components and therefore different activities.

MICs of ten plant polyphenols including castalagin and tannic acid, that influence the growth of pathogenic bacteria, were examined by Taguri et al. [20]. Among other bacterial species, several strains (23) of *E. coli* (both, pathogenic and non-pathogenic) served as test organisms. The average MICs for all *E. coli* strains of castalagin were 705 µg/mL and of tannic acid were 2073 µg/mL, which roughly coincides with MIC values of castalagin (292–883 µg/mL) obtained in this study. A significant difference in the determined MIC values of tannic acid, which are more than one order of magnitude higher than the MICs obtained in our study (32–200 µg/mL), was detected. MIC values of tannic acid, obtained in alternative previous studies [17,19] are also lower than the ones in the study by Taguri et al. [20]. Many factors must be considered when comparing the results of different studies. First of all, tannic acid is not a pure compound but a mixture of different tannins or even other substances and its composition may vary significantly between different sources [15]. Also factors like inoculum concentration (the concentration of *E. coli* in our study was one order of magnitude higher than in the study by Taguri et al. [20]) [27], MIC determination method applied [26], bacterial strain [20] and growth medium composition used play a role [28,29].

All studied samples exhibited promising antibacterial activity, some higher than the other, but MICs of both extracts and pure components are similar. This is especially beneficial when used as functional feed supplements, since their antimicrobial efficiencies are similar, but the extracts are more affordable and also contain other components that can improve and increase livestock production.

### 2.3. Influence of Growth Medium Concentration on MICs of Investigated Tannins against E. coli

**Figure 3:**
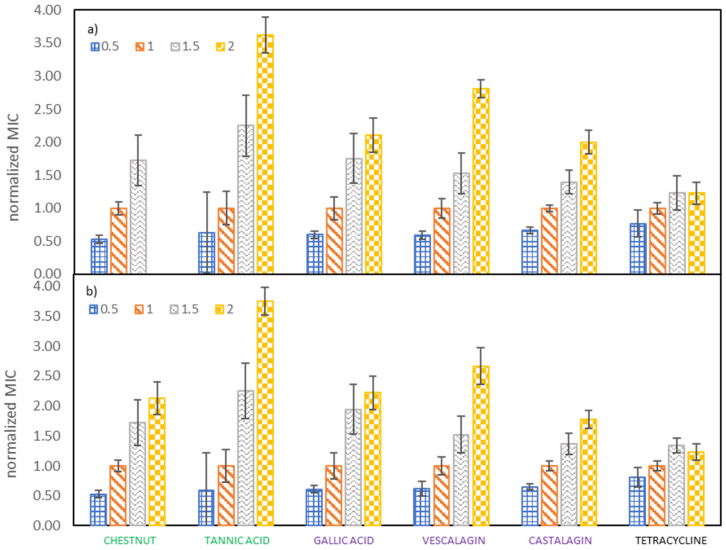
Dependence of the normalized MIC on the *E. coli* growth medium concentration, determined by (**a**) measuring OD and (**b**) using INT dye. MIC values were normalized with respect to the growth medium concentration recommended by the producer.

The important goal of our study was to determine the effect of growth medium strength on MIC values of investigated tannins against *E. coli*. Therefore, the growth medium concentration was increased from half to double the concentration recommended by the producer. From Figure 2 it can be observed that both the tested samples (vescalagin, castalagin, tannic acid, gallic acid, Colistizer, as well as chestnut, mimosa and quebracho extract) and the growth medium concentrations (that vary from half to double the concentration recommended by the producer) affect the MIC values. This is even more evident when we plot the normalized MIC values as a function of growth medium concentrations, like depicted in Figure 3 and Supplementary Material Figure S4, where MIC values are normalized with respect to the growth medium strength recommended by the producer. Average values with associated 95% confidence intervals are collected in Supplementary Material Tables S3 and S4. From Figure 3 it can be deducted that results obtained by both methods of determination are very similar and that growth medium strength plays an important role in antimicrobial activity. MIC values of all tested samples increase roughly linearly with the growth medium concentration. Additionally, results for the majority of pure compounds and extracts show, that when the recommended concentration of growth medium is doubled or halved, MIC values also double or halve, respectively. Slopes and correlation coefficients of the linear dependence of the normalized MIC on the growth medium concentration are reported in Table S5 (determination with OD method) and Table S6 (determination with INT dye). The largest deviation from this trend was detected for tannic acid, where the MIC value at the highest growth medium concentration is 3.75 of the MIC value at the growth medium concentration recommended by the producer. This may be partly ascribed to a lower MIC at the growth medium concentration recommended by the producer, according to which all values were normalized. Any experimental error at the growth medium concentration recommended by the producer will transfer to all other normalized values, as can be observed in Supplementary Material Figure S5, where all values were normalized with respect to the one and a half growth medium concentration recommended by the producer. However, although the trend for tannic acid is not the same as observed for the remaining pure compounds and extracts, the dependence of MIC on growth media concentration is even more pronounced. 

Tetracycline represents the only tested sample where only relatively weak dependence of MIC values on growth media strength was observed. While MIC is still increasing with rising growth media strength, the increase is not so profound. Namely, the MIC value at the lowest concentration (half the concentration recommended by the producer) being 0.82% of the MIC value at the concentration recommended by the producer and the MIC value at the highest concentration (double the concentration recommended by the producer) is only 44% higher than MIC value at the concentration recommended by the producer, although the amount of available nutrients has doubled. 

Explanations for this discrepancy can be sought in different mechanisms of action. In the case of tetracycline, an increase of media strength does not directly influence its mechanism of action, but rather provides the bacteria with better growth conditions, which enables them to survive at slightly higher concentrations of tetracycline. On the contrary, in the case of tannins doubling of MIC values when doubling the growth media concentration agrees well with the theory, that for their antibacterial activity a direct interaction with growth media constituents is responsible. Their main mechanism of action lies probably in chelation of microelements within the growth media (e.g., iron [17,18]). When the medium strength is doubled (the concentration of microelements is doubled) also the tannin concentration should be doubled in order to effectively chelate these microelements and consequently make them unavailable to bacteria. To confirm the theory, it would make sense to analyze the free ions after the addition of tannins to the varying concentrations of growth medium. Moreover, the interactions with essential proteins in the growth media may be responsible for the antimicrobial effectiveness of tannins as well, since tannins were also found to interact with proteins [40]. But the increased media strength could also affect the antibacterial activity of tannins in the opposite way. If there, in fact, existed an alternative mechanism of action, the stronger growth media would be able to form more complexes with tannins (either by proteins or by metal ions of the media) and thereby disable them from further action.

## 3. Materials and Methods 

### 3.1. Materials

Meat peptone and meat extract were purchased from Merck, Darmstadt, Germany. 2-(4-Iodophenyl)-3-(4-nitrophenyl)-5-phenyl-2*H*-tetrazolium chloride (INT), Tetracycline (≥98.0% (NT)) and Sephadex LH-20 were purchased from Sigma-Aldrich, St. Louis, MO, USA. All materials were used as received. 

### 3.2. Plant Extracts

For the study, three pure compounds were selected (gallic acid from Sigma-Aldrich, St. Louis, MO, USA; vescalagin; castalagin) as well as various plant extracts (chestnut extract named Farmatan from Tanin Sevnica, Sevnica, Slovenia; quebracho extract named Tannino Red Plus Polvere sacco from Tecnofood, Begoglio, Italy; mimosa extract named Tannino Codice M from Tecnofood, Begoglio, Italy; a sample containing tannic acid named Colistizer from Guangzhou Insighter, Guangdong, China, and tannic acid from Sigma-Aldrich, St. Louis, MO, USA, 96311-250G-F, Lot: BCBT8361). All purchased compounds were used as received. Castalagin and vescalagin were isolated from the chestnut extract following the procedure described in the next subsection. In the Supplementary Material Table S7 it can be observed, that extracts of quebracho, mimosa and Colistizer contain a very small percentage of gallic acid (<0.4%) and that they contain 70–80% of tannins.

### 3.3. Isolation of Vescalagin and Castalagin 

Vescalagin and castalagin were isolated from chestnut extract, where their initial contents are 6.0% and 4.1%, respectively, according to the producer. The detailed composition of the chestnut extract is accessible in Supplementary Material Table S8.

The process of isolation was slightly modified from the one described in the article of Moilanen and Salminen [15]. Several parallel purifications were performed using the following procedure: 30 mL of water/acetone mixture (70/30, *V*/*V*) was added to 15 g of Farmatan powder, sonicated for 15 min at 25 °C and filtered subsequently. The obtained solution was applied on a Sephadex LH-20 column. The column was first washed with water. Desired tannins were then eluted from the column using 30% aq. methanol solution, 50% aq. methanol solution, and 10% aq. acetone solution. The mobile phase composition was subsequently gradually changed to a 75% aq. acetone solution to wash the stationary phase. Finally, the column was first equilibrated by 50% aq. methanol, followed by pure water and then reused for the next separation. 30–50% aq. methanol eluents and 10% aq. acetone eluents were collected in smaller, 50 mL fractions, in order to successfully separate vescalagin and castalagin. Their content in each fraction was carefully monitored using HPLC. All vescalagin rich as well as all castalagin rich fractions were joined and the solvent was evaporated. Both vescalagin and castalagin rich samples were dissolved in water again and applied on Sephadex LH-20 column for the second time, respectively. Afterwards, the purest fractions were joined and solvents evaporated again. The whole procedure was repeated until the sufficient amount of partially purified sample was collected for further purification.

The obtained vescalagin rich as well as castalagin rich fractions were further purified by preparative HPLC. They were dissolved in type 1 water (0.8 g in 5 mL of water), filtered through a 0.2 μm PTFE syringe filter and applied on the preparative HPLC column filled with a Merck Li-Chroprep RP-18 (44 × 3.7 cm i.d., 40–63 μm); elution was performed with methanol/water (0/100—80/20, *V*/*V*), acidified by formic acid (1% *V*/*V*). The HPLC system consisted of a Waters 2535 Quaternary Gradient Modul (Waters Corp. Milford, MA, USA), a Waters 2998 photodiode array Detector (Waters Corp. Singapore), and a Waters Fraction Collector III (Waters Corp. Shinagawa City, Japan) [41]. The purity of fractions was checked using UPLC-DAD chromatography. The organic solvent was evaporated from pure enough fractions and the solid material was obtained by lyophilization. The final purification was conducted on a semi-preparative C18 chromatographic column (150 × 21.20 mm, Gemini^®^ 10 mm, C-18, 110 Å, Axia packed, Phenomenex), this separation was performed using a 0.1% aqueous solution of formic acid as mobile phase A and 0.1% methanolic solution of formic acid as mobile phase B. At the beginning of the separation, only mobile phase A was used and afterwards the content of mobile phase B was gradually increased to 60%. The purity of fractions was again examined using UPLC-DAD chromatography. The organic solvent was evaporated and the final product was obtained by lyophilization. Its purity was checked by LC-MS.

### 3.4. Chromatographic Techniques

#### 3.4.1. High-Performance Liquid Chromatography (HPLC)

HPLC system (FLCXR Shimadzu (Shimadzu corp., Kyoto, Japan)) with DAD detector (SPD-M20A) using Kinetex 2.6u C18 100 A column of 100 mm × 4.60 mm dimension (Phenomenex, Torrance, CA, USA) was applied. Two mobile phases were used, 0.1% solution of phosphoric acid in water (solvent A) and in methanol (solvent B). The separation started with using only the solvent A as a mobile phase and then gradually increasing the percentage of the solvent B in the mobile phase.

#### 3.4.2. Ultra-Performance Liquid Chromatography (UPLC)

After each preparative or semi-preparative purification step, the resulting fractions were analysed by UPLC-DAD. Sample analyses was carried out with an Acquity UPLC system (Waters Corporation, Milford, MA, USA) coupled with a DAD detector. The separation was performed on 100 × 2.1 mm i.d., 1.7 μm, Acquity UPLC BEH Phenyl column (Waters Corporation, Wexford, Ireland). The eluent flow rate was set to 0.5 mL/min. The elution profile used two solvents, acetonitrile (A) and 0.1% aqueous formic acid (B): 0–0.5 min, 0.1% A in B; 0.5–5.0 min, 0.1–30% A in B (linear gradient); 5.0–5.1 min, 30–90% A in B (linear gradient); 5.1–8.5 min, column wash and stabilization. HPLC/UV chromatogram was collected for the first 6 min of the separation.

#### 3.4.3. Liquid Chromatography-Mass Spectrometry (LC-MS)

The mass spectroscopy analyses was performed using an Aquity UPLC system (Waters Corp., Milford, MA, USA) coupled with a quadrupole-Orbitrap mass spectrometer (Q ExactiveTM, Thermo Fischer Scientific GmbH, Bremen, Germany). The separation was carried out on 1.7 μm, Acquity UPLC BEH Phenyl 1.7 μm, 2.1 × 30 mm column (Waters Corporation, Wexford, Ireland). The elution profile was as follows: 0–0.1 min 3% A in B (isocratic); 0.1–3.0 min, 3–45% A in B (linear gradient); 3.0–4.2 min, column wash, and stabilization. The UV (λ = 190–500 nm) and MS data (detection in negative mode) were collected. A heated ESI source (H-ESI II, Thermo Fischer Scientific GmbH) with the following setup was used: spray voltage, −3.0 kV; capillary temperature, 380 °C; sheat, aux and sweep gas (N_2_) flow rate 60, 20, and 0 arbitary units, respectively. The mass range of the Orbitrap detector was set to the *m/z* 200–3000, while the resolution was set at 70,000. The automatic gain of 3 × 10^6^ was used.

### 3.5. Preparation of Inoculum

The in vitro antibacterial activity of the tannin extracts and pure compounds was assayed against *Escherichia coli* K12 (DSM 498, Leibniz Institute, DSMZ-German Collection of Microorganisms and Cell Cultures GmbH). The bacterial culture was prepared according to the CLSI standard [42]. Bacteria were precultured in the nutrient broth (5 g of meat peptone and 3 g of meat extract in 1 L of deionized water at pH 7, which is the concentration of a broth recommended by the producer) for 3–4 h in a rotary shaker at 37 °C. The turbidity of broth culture was adjusted to the concentration of 1 × 10^8^ CFU/mL using a spectrophotometer (Carry 50 UV-Vis Spectrophotometer, Agilent Technologies) and diluted to the final inoculum concentration of about 1 × 10^5^ CFU/mL. Colony forming units were verified with the spread-plate method.

### 3.6. Antimicrobial Activity

To evaluate the antimicrobial activity MICs of investigated samples against *E. coli* were determined with broth microdilution assay using 96-well microplates and readings were evidenced by two complementary methods—by measuring optical density (OD) or by adding iodonitrotetrazolium chloride (INT) dye irrespectively. MIC represents the lowest concentration of an antimicrobial agent that prevents the growth of microorganisms.

The concentration of nutrients in the growth medium was varied by changing the broth concentration from half to double the concentration recommended by the producer.

Samples were dissolved in the growth medium. Each sample was prepared in seven densely descending concentrations, that were applied to the microplate. Bacterial culture was subsequently added to the wells. The final volume in each well was 200 µL. A negative control (control of growth) and a positive control, where tetracycline was tested as a reference antibiotic, were performed. The microplates were inserted in the spectrophotometer (Tecan Infinite F200) and incubated at 37 °C for 20 h. Afterwards, MICs were determined by the two methods. Experiments were carried out in six runs and MICs were determined as the average of the replicates [43].

#### 3.6.1. Antibacterial MIC Determination by Measuring OD 

During incubation bacterial growth was followed by measuring the OD with a spectrophotometer (Tecan Infinite F200) using Magellan software. Measurements were made at the wavelength 595 nm every 5 min and prior to every measurement, 10 s stirring was applied.

OD of the background was subtracted from the total OD to obtain only the bacterial turbidity. Dependency graphs of OD vs. time were plotted and the lowest concentration of tannins with no bacterial growth detected, was determined as MIC [29]. If the OD values after 20 h of incubation were lower than 0.05, it was determined that bacterial growth was not present.

#### 3.6.2. Antibacterial MIC Determination Using INT

2-(4-Iodophenyl)-3-(4-nitrophenyl)-5-phenyl-2*H*-tetrazolium chloride (INT) was applied as a growth indicator. For MIC determination with INT, 20 µL of 2000 µg/mL INT, dissolved in water, was added to each well on a microplate after 20-hour incubation. The microplate was then placed into a rotary shaker at 37 °C for about 30 min in the dark. In the presence of respiratory activity, soluble INT reduces to insoluble violet formazan dye. The color change is fast and formazan, formed from INT, is stable, so that the color does not fade. MICs were determined as the lowest concentrations of tannin extracts or pure compounds without the presence of red-violet precipitate [44,45,46].

## 4. Conclusions

Vescalagin and castalagin with high purity were successfully isolated from the chestnut extract. MIC values of several tannin extracts and pure compounds were determined against *E. coli* using either measurement of OD or the addition of INT dye and both methods provided reproducible and similar results. The findings of the present study show that all studied samples possess promising antimicrobial activities. Tannic acid represents the best inhibitor of bacterial growth whereas gallic acid is the least effective. Castalagin and vescalagin provide somewhat lower MICs than the chestnut extract, the sample they were isolated from. Consequently, they contribute significantly, but not exclusively, to the antimicrobial activity of the chestnut extract. Moreover, MIC values depend significantly on the growth media concentration. With the rise in the growth medium concentration, MICs of all studied samples increase roughly linearly while the relative order of MIC values of all tested samples remains the same. With the doubled (or halved) concentration of the growth medium, MIC values also double (or halve), confirming the assumption that direct interactions of tannins with growth medium nutrients play a crucial role in their antimicrobial activity. However, to show further generalization of dependence of MIC values on growth media strength, additional studies using various bacterial species and strains are needed, including representatives of Gram-positive bacteria. Furthermore, complementary studies of metal ions chelation by tannins will also be performed to confirm the proposed mechanism of action. Last but not least, alternative growth media shall be investigated as well. In order to plan the further application of tannins or in vivo tests of their activity, a detailed knowledge of the environment (e.g., feed composition) in which tannins are going to act is, therefore, of utmost importance for the successful prevention of bacterial growth. 

## Supplementary Materials

The following are available online at https://www.mdpi.com/1420-3049/25/12/2947/s1, Figures S1 and S2: 1H NMR spectra of vescalagin and castalagin, Figure S3: A comparison of MICs determined by different methods, Figure S4: Dependence of normalized MICs on the growth medium concentration, Tables S1–S4: MIC values of tannins in differently concentrated *E. coli* growth media, Tables S5 and S6: Slopes and correlation coefficients of the obtained linear regression curves, Figure S5: Dependence of differently normalized MIC on the concentration of *E. coli* growth media, Table S7: The percentage of tannins and the gallic acid content in the extracts, Table S8: Composition of the chestnut extract.

## References

[1] (2003). EC (1831/2003) Regulation (EC) No 1831/2003 of the European Parliament and of the Council of 22 September 2003 on additives for use in animal nutrition. Off. J. Eur. Union.

[2] Redondo L.M., Chacana P.A., Dominguez J.E., Fernandez Miyakawa M.E. (2014). Perspectives in the use of tannins as alternative to antimicrobial growth promoter factors in poultry. Front. Microbiol..

[3] Oliver S.P., Murinda S.E., Jayarao B.M. (2011). Impact of Antibiotic Use in Adult Dairy Cows on Antimicrobial Resistance of Veterinary and Human Pathogens: A Comprehensive Review. Foodborne Pathog. Dis..

[4] Chattopadhyay M.K. (2014). Use of antibiotics as feed additives: A burning question. Front. Microbiol..

[5] Marshall B.M., Levy S.B. (2011). Food Animals and Antimicrobials: Impacts on Human Health. Clin. Microbiol. Rev..

[6] Michael C.A., Dominey-Howes D., Labbate M. (2014). The Antimicrobial Resistance Crisis: Causes, Consequences, and Management. Front. Public Health.

[7] Mathur S., Singh R. (2005). Antibiotic resistance in food lactic acid bacteria—A review. Int. J. Food Microbiol..

[8] Windisch W., Schedle K., Plitzner C., Kroismayr A. (2008). Use of phytogenic products as feed additives for swine and poultry. J. Anim. Sci..

[9] Huang Q., Liu X., Zhao G., Hu T., Wang Y. (2018). Potential and challenges of tannins as an alternative to in-feed antibiotics for farm animal production. Anim. Nutr..

[10] Fraga-Corral M., García-Oliveira P., Pereira A.G., Lourenço-Lopes C., Jimenez-Lopez C., Prieto M.A., Simal-Gandara J. (2020). Technological application of tannin-based extracts. Molecules.

[11] Serrano J., Puupponen-Pimiä R., Dauer A., Aura A.-M., Saura-Calixto F. (2009). Tannins: Current knowledge of food sources, intake, bioavailability and biological effects. Mol. Nutr. Food Resour..

[12] Cowan M.M. (1999). Plant Products as Antimicrobial Agents. Clin. Microbiol. Rev..

[13] (1999). Plant Polyphenols 2: Chemistry, Biology, Pharmacology, Ecology.

[14] Khanbabaee K., Ree T. (2001). Van Tannins: Classification and Definition. R. Soc. Chem..

[15] Salminen J.-P., Karonen M. (2011). Chemical ecology of tannins and other phenolics: We need a change in approach. Funct. Ecol..

[16] Chung K.-T., Wong T.Y., Wei C.-I., Huang Y.-W., Lin Y. (1998). Tannins and Human Health: A Review. Crit. Rev. Food Sci. Nutr..

[17] Chung K.-T., Lu Z., Chou M.W. (1998). Mechanism of Inhibition of Tannic Acid and Related Compounds on the Growth of Intestinal Bacteria. Food Chem. Toxicol..

[18] Chung K.-T., Stevens Jr S.E., Lin W.-F., Wei C.I. (1993). Growth inhibition of selected food-borne bacteria by tannic acid, propyl gallate and related compounds. Lett. Appl. Microbiol..

[19] Henis Y., Tagari H., Volcani R. (1964). Effect of Water Extracts of Carob Pods, Tannic Acid, and Their Derivatives on the Morphology and Growth of Microorganisms. J. Appl. Microbiol..

[20] Taguri T., Tanaka T., Kouno I. (2004). Antimicrobial Activity of 10 Different Plant Polyphenols against Bacteria Causing Food-Borne Disease. Biol. Pharm. Bull..

[21] Borges A., Ferreira C., Saavedra M.J., Simões M. (2013). Antibacterial activity and mode of action of ferulic and gallic acids against pathogenic bacteria. Microbial Drug Resist..

[22] Saxena G., McCutcheon A.R., Farmer S., Towers G.H.N., Hancock R.E.W. (1994). Antimicrobial constituents of Rhus glabra. J. Ethnopharmacol..

[23] Yamanaka F., Hatano T., Ito H., Taniguchi S., Takanashi E., Okamoto K. (2008). Antibacterial Effects of Guava Tannins and Related Polyphenols on Vibrio and Aeromonas Species. Nat. Prod. Commun..

[24] Shuaibu M.N., Wuyep P.A., Yanagi T., Hirayama K., Tanaka T., Kouno I. (2008). The use of microfluorometric method for activity-guided isolation of antiplasmodial compound from plant extracts. Parasitol. Res..

[25] Becker H., Scher J.M., Speakman J.-B., Zapp J. (2005). Bioactivity guided isolation of antimicrobial compounds from Lythrum salicaria. Fitoterapia.

[26] Jiang L. (2009). Comparison of Disk Diffusion, Agar Dilution, and Uroth Microdilution for Antimicrobial Susceptibility Testing of Five Chitosans. Master’s Thesis.

[27] Gehrt A., Peter J., Pizzo P.A., Walsh T.J. (1995). Effect of Increasing Inoculum Sizes of Pathogenic Filamentous Fungi on MICs of Antifungal Agents by Broth Microdilution Method. J. Clin. Microbiol..

[28] Meletiadis J., Meis J.F.G.M., Mouton J.W., Verweij P.E. (2001). Analysis of Growth Characteristics of Filamentous Fungi in Different Nutrient Media. J. Clin. Microbiol..

[29] Balouiri M., Sadiki M., Ibnsouda S.K. (2016). Methods for in vitro evaluating antimicrobial activity: A review. J. Pharm. Anal..

[30] Scalbert A. (1991). Antimicrobial properties of tannins. Phytochemistry.

[31] Chu E.H.Y., Arbor A., Hollaender M.A., Nicoletti B., Kopits S.E., Ascani E., Mckusick V.A., Sutherland B.M., Woodhead A.D., Harling O.K., Hemingway R.W., Laks P.E. (1991). Plant Polyphenols: Synthesis, Properties, Significance.

[32] Ikigai H., Nakae T., Hara Y., Shimamura T. (1993). Bactericidal catechins damage the lipid bilayer. Biochim. Biophys. Acta.

[33] Anderson R.C., Vodovnik M., Min B.R., Pinchak W.E., Krueger N.A., Harvey R.B., Nisbet D.J. (2012). Bactericidal effect of hydrolysable and condensed tannin extracts on Campylobacter jejuni in vitro. Folia Microbiol..

[34] Akiyama H., Fujii K., Yamasaki O., Oono T., Iwatsuki K. (2001). Antibacterial action of several tannins against Staphylococcus aureus. J. Antimicrob. Chemother..

[35] Doern G.V., Tubert T.A., Chapin K., Rinaldi M.G. (1986). Effect of Medium Composition on Results of Macrobroth Dilution Antifungal Susceptibility Testing of Yeasts. J. Clin. Microbiol..

[36] Grossman T.H. (2016). Tetracycline antibiotics and resistance. Cold Spring Harb. Perspect. Med..

[37] Casewell M., Friis C., Marco E., McMullin P., Phillips I. (2003). The European ban on growth-promoting antibiotics and emerging consequences for human and animal health. J. Antimicrob. Chemother..

[38] Brglez Mojzer E., Knez Hrnčič M., Škerget M., Knez Ž., Bren U. (2016). Polyphenols: Extraction Methods, Antioxidative Action, Bioavailability and Anticarcinogenic Effects. Molecules.

[39] Min B.R., Pinchak W.E., Anderson R.C., Callaway T.R. (2007). Effect of Tannins on the In Vitro Growth of Escherichia coli O157:H7 and In Vivo Growth of Generic Escherichia coli Excreted from Steers. J. Food Prot..

[40] Karonen M., Oraviita M., Mueller-Harvey I., Salminen J.P., Green R.J. (2019). Ellagitannins with Glucopyranose Cores Have Higher Affinities to Proteins than Acyclic Ellagitannins by Isothermal Titration Calorimetry. J. Agric. Food Chem..

[41] Leppä M.M., Karonen M., Tähtinen P., Engström M.T., Salminen J.P. (2018). Isolation of chemically well-defined semipreparative liquid chromatography fractions from complex mixtures of proanthocyanidin oligomers and polymers. J. Chromatogr. A.

[42] CLSI (2012). Methods for Dilution Antimicrobial Susceptibility Tests for Bacteria That Grow Aerobically: Approved Standard.

[43] Wiegand I., Hilpert K., Hancock R.E.W. (2008). Agar and broth dilution methods to determine the minimal inhibitory concentration (MIC) of antimicrobial substances. Nat. Protoc..

[44] Eloff J.N. (2019). Avoiding pitfalls in determining antimicrobial activity of plant extracts and publishing the results. BMC Complement. Altern. Med..

[45] Klančnik A., Piskernik S., Jeršek B., Smole Možina S. (2010). Evaluation of diffusion and dilution methods to determine the antibacterial activity of plant extracts. J. Microbiol. Methods.

[46] Eloff J.N. (1998). A sensitive and quick microplate method to determine the minimal inhibitory concentration of plant extracts for bacteria. Planta Med..

