# Supplementary material for: The Effect of Growth Medium Strength on Minimum Inhibitory Concentrations of Tannins and Tannin Extracts against E. coli
Source: Molecules. 2020 Jun 26;25(12):2947. doi: 10.3390/molecules25122947 (PMC7355419; doi:10.3390/molecules25122947)
Supplement: Supplementary file 1 [file molecules-25-02947-s001.pdf]

Molecules

Supplementary Material

The Effect of Growth Medium Strength on Minimum Inhibitory Concentrations of Tannins and Tannin Extracts against *E. coli*

Sara Štumpf [1], Gregor Hostnik [1], Mateja Primožič [1], Maja Leitgeb [1,2], Juha-Pekka Salminen [3] and Urban Bren [1,4,*]

[1] Faculty of Chemistry and Chemical Technology, University of Maribor, 2000 Maribor, Slovenia; sara.stumpf@um.si (S.Š.); gregor.hostnik@um.si (G.H.); mateja.primozic@um.si (M.P.); maja.leitgeb@um.si (M.L.)
[2] Faculty of Medicine, University of Maribor, 2000 Maribor, Slovenia
[3] Natural Chemistry Research Group, Department of Chemistry, University of Turku, 20014 Turku, Finland; j-p.salminen@utu.fi
[4] Faculty of Mathematics, Natural Sciences and Information Technologies, University of Primorska, 6000 Koper, Slovenia
* Correspondence: urban.bren@um.si; Tel.: +386-2-2294-421

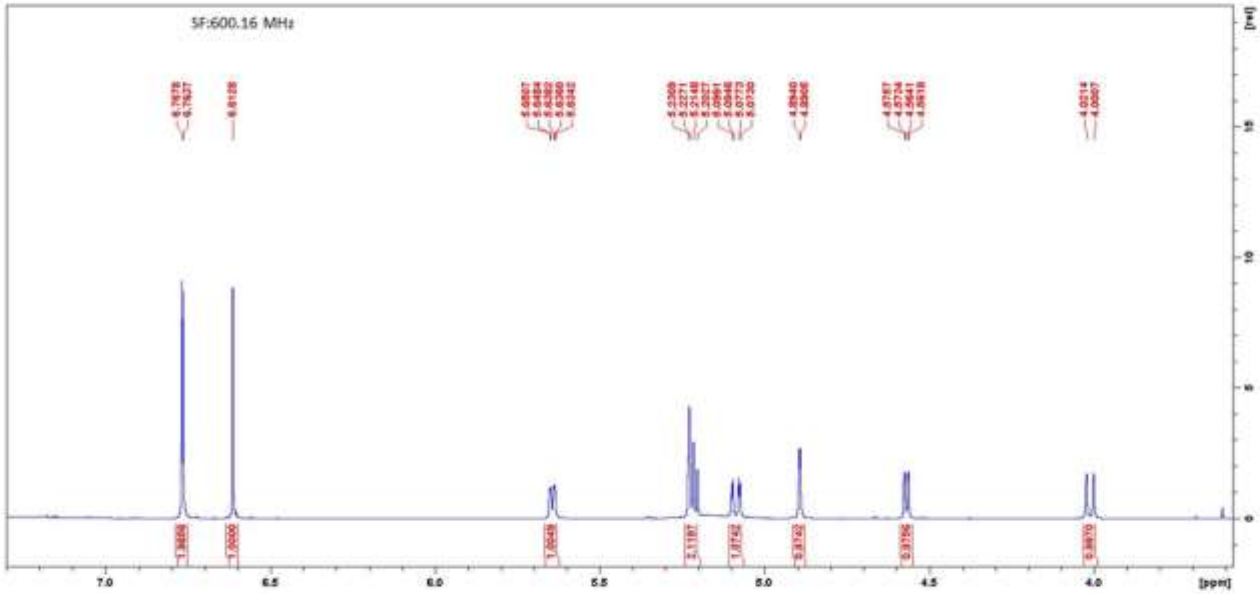

Figure S1. 1H NMR spectrum of vescalagin.

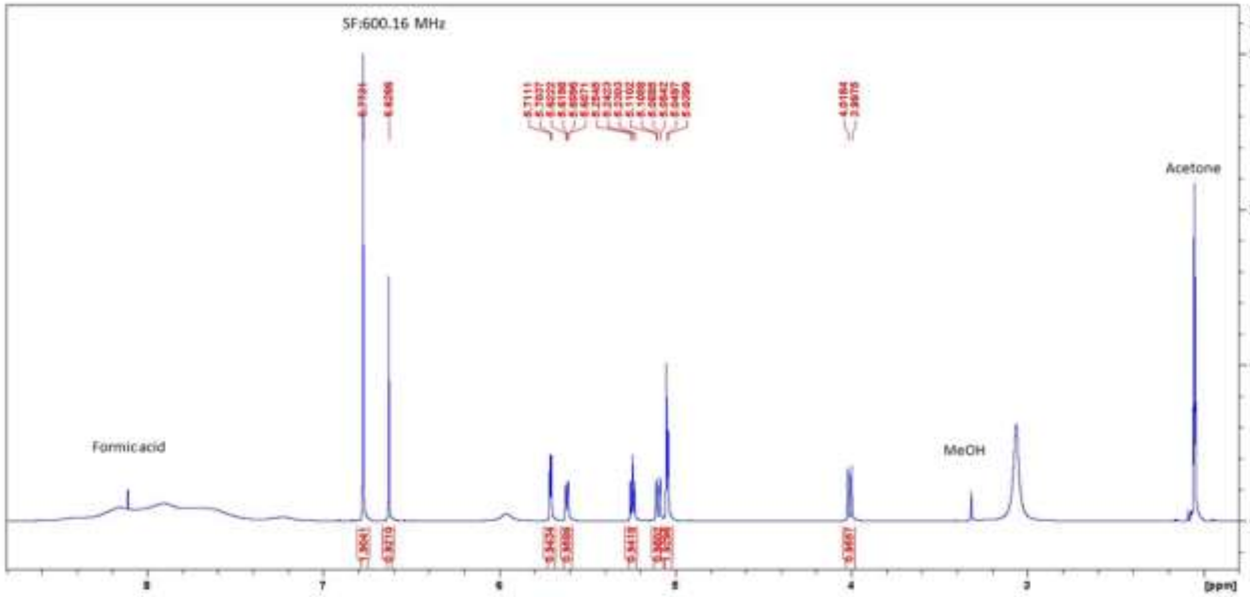

Figure S2. 1H NMR spectrum of castalagin.

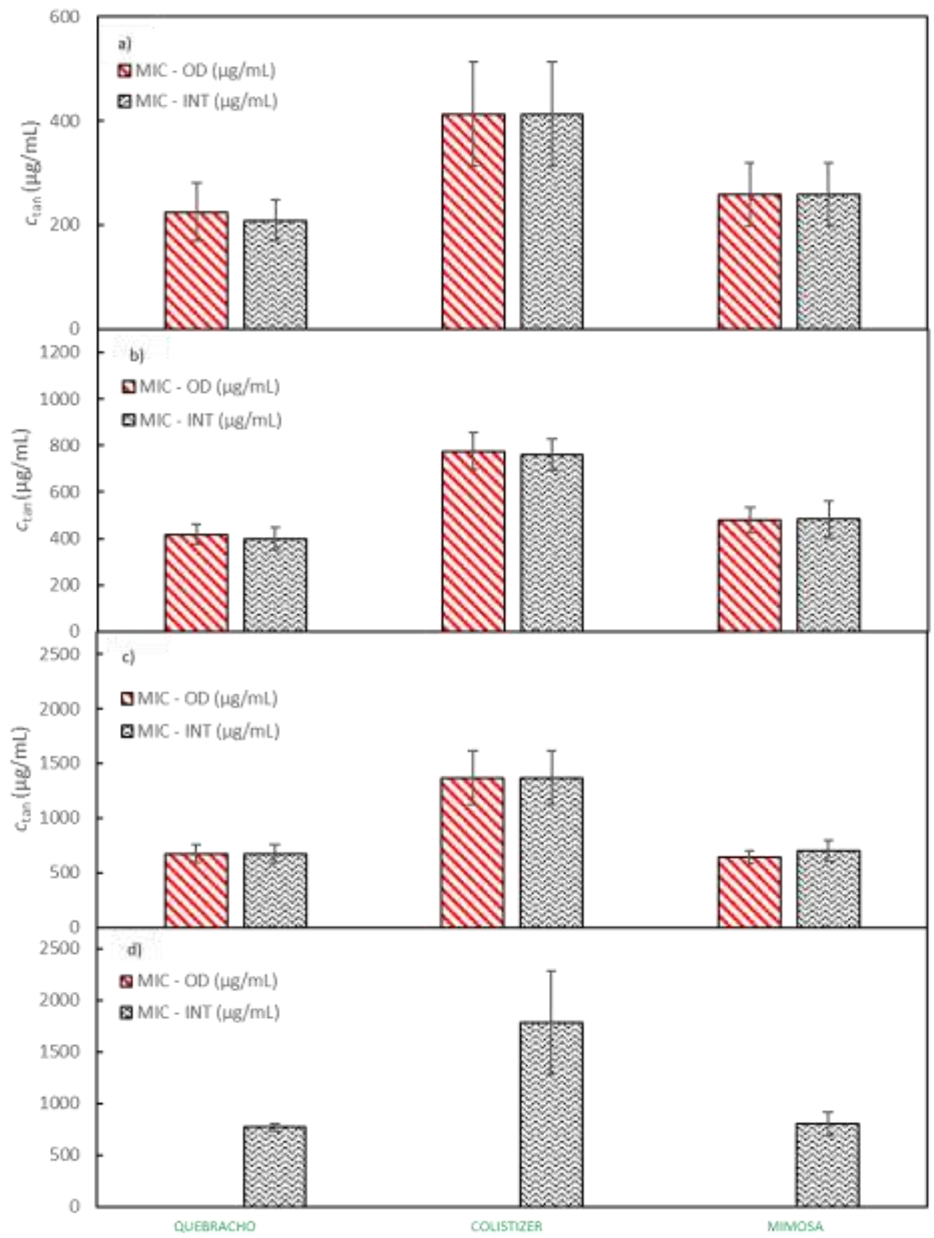

Figure S3. A comparison of MIC values for crude commercial extracts of quebracho, mimosa and Colistizer, determined by measurement of the OD and by addition of the INT dye at a) half the concentration of E. coli growth medium recommended by the producer, b) the concentration of E. coli growth medium recommended by the producer, c) one and a half the concentration of E. coli growth medium recommended by the producer and d) double the concentration of E. coli growth medium recommended by the producer. Error bars represent 95% confidence interval.

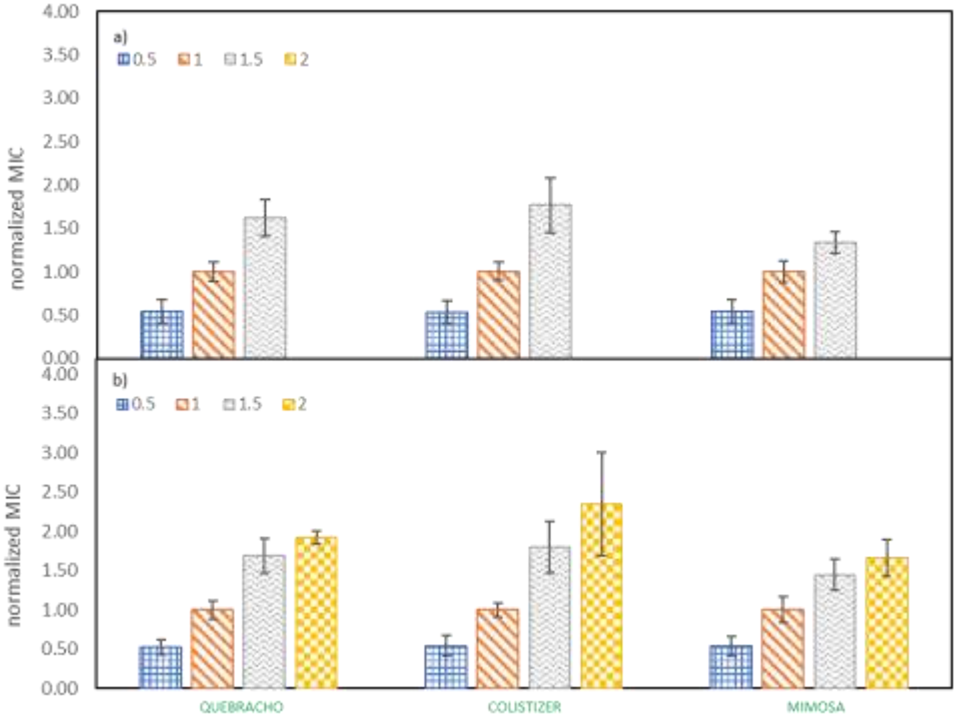

Figure S4. Dependence of the normalized MIC on the E. coli growth medium concentration for crude commercial extracts of quebracho, mimosa and Colistizer, determined by a) measuring OD and b) using INT dye. MIC values were normalized with respect to the growth medium concentration recommended by the producer.

Table S1. Mean values of MICs (µg/mL) of tannins in differently concentrated growth media determined by the measurement of OD. A 95% confidence interval is added.

	0.5 × concentrated medium	1 × concentrated medium	1.5 × concentrated medium	2 × concentrated medium
Chestnut extract	300 ± 33	567 ± 54	975 ± 217	/
Quebracho extract	225 ± 55	417 ± 43	675 ± 86	/
Colistizer	413 ± 100	775 ± 80	1367 ± 245	/
Mimosa extract	258 ± 61	480 ± 56	640 ± 57	/
Tannic acid	33 ± 35	53 ± 14	120 ± 25	193 ± 29
Gallic acid	567 ± 54	950 ± 163	1667 ± 359	2000 ± 248
Vescalagin	208 ± 21	350 ± 52	533 ± 108	983 ± 46
Castalagin	292 ± 21	442 ± 21	617 ± 79	883 ± 79
Tetracycline	0.29 ± 0.08	0.38 ± 0.03	0.47 ± 0.1	0.47 ± 0.06

Table S2. Mean values of MICs (µg/mL) of tannins in differently concentrated growth media determined by the addition of INT dye. A 95% confidence interval is added.

	0.5 × concentrated medium	1 × concentrated medium	1.5 × concentrated medium	2 × concentrated medium
Chestnut extract	300 ± 33	567 ± 54	975 ± 217	1210 ± 155
Quebracho extract	208 ± 39	400 ± 47	675 ± 86	770 ± 34
Colistizer	413 ± 100	760 ± 68	1367 ± 245	1783 ± 502
Mimosa extract	258 ± 61	483 ± 79	700 ± 94	800 ± 113
Tannic acid	32 ± 33	53 ± 14	120 ± 25	200 ± 25
Gallic acid	550 ± 57	900 ± 199	1750 ± 373	2000 ± 248
Vescalagin	217 ± 43	350 ± 52	533 ± 108	933 ± 108
Castalagin	292 ± 21	450 ± 33	617 ± 79	800 ± 66
Tetracycline	0.32 ± 0.06	0.39 ± 0.03	0.52 ± 0.05	0.48 ± 0.05

Table S3. Normalized MICs of tannins in differently concentrated growth medium determined by the measurement of optical density. MIC values were normalized according to the concentration of the growth medium recommended by the producer. A 95% confidence interval is added.

	0.5 × concentrated medium	1 × concentrated medium	1.5 × concentrated medium	2 × concentrated medium
Chestnut extract	0.53 ± 0.06	1 ± 0.1	1.72 ± 0.38	/
Quebracho extract	0.54 ± 0.14	1 ± 0.11	1.62 ± 0.21	/
Colistizer	0.53 ± 0.13	1 ± 0.11	1.76 ± 0.32	/
Mimosa extract	0.54 ± 0.14	1 ± 0.12	1.33 ± 0.12	/
Tannic acid	0.63 ± 0.62	1 ± 0.25	2.25 ± 0.47	3.63 ± 0.27
Gallic acid	0.60 ± 0.06	1 ± 0.17	1.75 ± 0.38	2.11 ± 0.26
Vescalagin	0.60 ± 0.06	1 ± 0.15	1.52 ± 0.31	2.81 ± 0.13
Castalagin	0.66 ± 0.05	1 ± 0.05	1.4 ± 0.18	2 ± 0.18
Tetracycline	0.77 ± 0.2	1 ± 0.09	1.23 ± 0.26	1.23 ± 0.17

Table S4. Normalized MICs of tannins in differently concentrated growth media determined by the addition of the INT dye. MIC values were normalized according to the concentration of the growth medium recommended by the producer. A 95% confidence interval is added.

	0.5 × concentrated medium	1 × concentrated medium	1.5 × concentrated medium	2 × concentrated medium
Chestnut extract	0.53 ± 0.06	1 ± 0.10	1.72 ± 0.38	2.14 ± 0.27
Quebracho extract	0.52 ± 0.10	1 ± 0.12	1.69 ± 0.22	1.93 ± 0.09
Colistizer	0.54 ± 0.13	1 ± 0.09	1.8 ± 0.32	2.35 ± 0.66
Mimosa extract	0.53 ± 0.13	1 ± 0.16	1.45 ± 0.19	1.66 ± 0.23
Tannic acid	0.59 ± 0.62	1 ± 0.27	2.25 ± 0.47	3.75 ± 0.23
Gallic acid	0.61 ± 0.06	1 ± 0.22	1.94 ± 0.41	2.22 ± 0.28
Vescalagin	0.62 ± 0.12	1 ± 0.15	1.52 ± 0.31	2.67 ± 0.31
Castalagin	0.65 ± 0.05	1 ± 0.08	1.37 ± 0.18	1.78 ± 0.15
Tetracycline	0.82 ± 0.16	1 ± 0.08	1.34 ± 0.12	1.24 ± 0.14

Table S5. Slope and correlation coefficient of the linear regression curve obtained with the OD method.

	slope	R^2
CHESTNUT	1.19	0.986
QUEBRACHO	1.08	0.993
COLISTIZER	1.23	0.981
MIMOSA	0.80	0.991
TANNIC ACID	2.05	0.950
GALLIC ACID	1.06	0.980
VESCALAGIN	1.43	0.923
CASTALAGIN	0.88	0.981
TETRACYCLINE	0.32	0.890

Table S6. Slope and correlation coefficient of the linear regression curve obtained with the INT method.

	slope	R^2
CHESTNUT	1.11	0.990
QUEBRACHO	0.98	0.971
COLISTIZER	1.24	0.990
MIMOSA	0.76	0.974
TANNIC ACID	2.14	0.948
GALLIC ACID	1.16	0.955
VESCALAGIN	1.33	0.934
CASTALAGIN	0.75	0.999
TETRACYCLINE	0.32	0.769

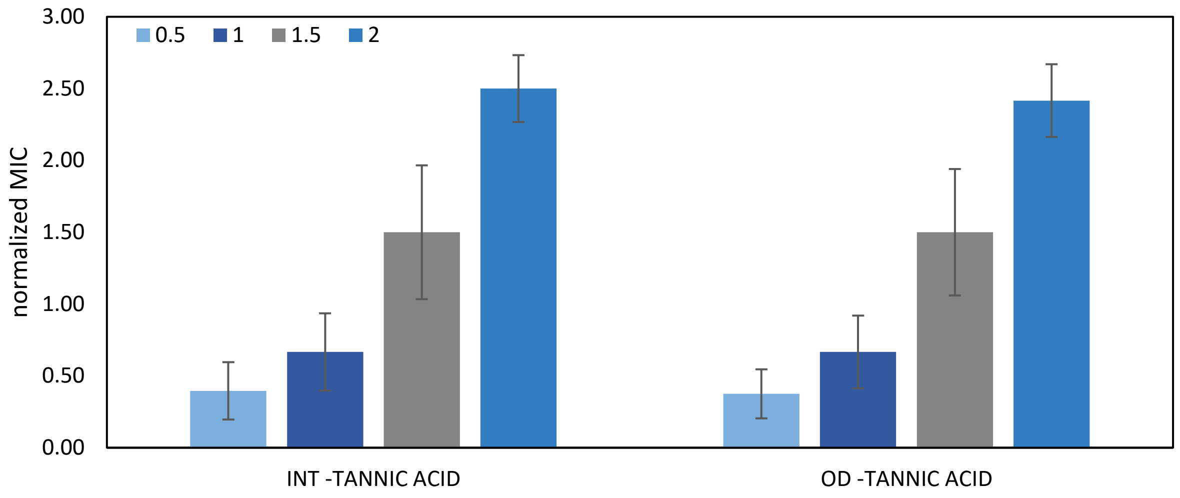

Figure S5. Dependence of normalized MIC on the concentration of E. coli growth media, determined by the addition of INT dye and with the OD measurement. MIC values are normalized according to one and a half the concentration of E. coli growth medium recommended by the producer.

Table S7. The percentage of tannins in different extracts, determined by *Quantitative analysis of tanning agents by filter method* (International standard ISO 14088) and the content of gallic acid in different extracts.

extract	Tannin content (%)	Gallic acid content (%)
mimosa	72.5	0.03
quebracho	79.8	0.37
Colistizer	74.5	0.27

Table S8. Composition of chestnut extract.

component	content (%)
vescalin	1.0
castalin	1.6
roburin A	2.0
gallic acid	2.5
roburin B/C	1.3
grandinin	0.5
roburin D	1.0
vescalagin	6.0
roburin E	2.2
castalagin	4.1
ellagic acid	0.6
water	8.1
unidentified	69.1
